# Autonomic Neuropathy—a Prospective Cohort Study of Symptoms and *E*/*I* Ratio in Normal Glucose Tolerance, Impaired Glucose Tolerance, and Type 2 Diabetes

**DOI:** 10.3389/fneur.2018.00154

**Published:** 2018-03-14

**Authors:** Malin Zimmerman, Kaveh Pourhamidi, Olov Rolandsson, Lars B. Dahlin

**Affiliations:** ^1^Hand Surgery, Department of Translational Medicine, Lund University, Skåne University Hospital, Malmö, Sweden; ^2^Department of Hand Surgery, Skåne University Hospital, Malmö, Sweden; ^3^Family Medicine, Department of Public Health and Clinical Medicine, Umeå University, Umeå, Sweden

**Keywords:** diabetes mellitus type 2, glucose intolerance, autonomic nervous system diseases, autonomic nervous system, diabetic neuropathies, glycosylated hemoglobin A, glucose tolerance test, disease progression

## Abstract

**Background:**

Autonomic neuropathy in diabetes, in addition to causing a range of symptoms originating from the autonomic nervous system, may increase cardiovascular morbidity. Our aim was to study the progression of autonomic neuropathy, based on symptom score and evaluation of an autonomic test, in persons with normal and impaired glucose tolerance and in patients with type 2 diabetes (T2D).

**Methods:**

Participants were recruited in 2003/2004 with a follow-up in 2014. The participants’ glucose tolerance was categorized using oral glucose tolerance tests. Symptoms were evaluated using an autonomic symptom score (ASS), ECG was used to test cardiac autonomic function based on the expiration/inspiration ratio (*E*/*I* ratio), and blood samples were taken on both occasions.

**Results:**

ASSs were higher at follow-up in the T2D patients than in the normal glucose tolerance group (mean 1.21 ± 1.30 vs. 0.79 ± 0.7; *p* < 0.05). *E*/*I* ratio did not deteriorate more than could be expected as an aging effect in well-controlled T2D. No relationship was found between *E*/*I* ratio and HbA1c or ASS.

**Conclusion:**

The presence of autonomic symptoms increased over time in T2D patients, but the symptoms did not correlate with the *E*/*I* ratio in this metabolically well-controlled cohort. ASSs can be a useful clinical tool when assessing the progression of autonomic dysfunction in patients with abnormal glucose metabolism.

## Introduction

The rising global incidence of diabetes mellitus means that an increasing number of people are living with complications from diabetes (1). Neuropathy, known to occur in 30–40% of patients with diabetes, can be divided into peripheral and autonomic neuropathy. Autonomic neuropathy causes difficulties in adapting to changes in posture or activity level, erectile dysfunction, incontinence, gastrointestinal disturbances, and an increased risk of cardiovascular morbidity (2). At present, available treatments are aimed at secondary prevention, including strict glucose control and lifestyle modifications (3).

In a small recent study on type 1 diabetes (T1D), an increase in HbA1c was related to impaired cardiovascular autonomic function (3), which correlates well with the results of a number of large studies that have shown beneficial effects from strict glucose control on cardiac autonomic neuropathy (CAN) in patients with T1D (4). In the DCCT trial, intensive glucose control prevented the development of abnormal heart rate variability (5). One previous study has shown abnormal expiration/inspiration ratio (*E*/*I* ratio) in 35% of the studied population of patients with type 2 diabetes (T2D) (6), and a large trial demonstrated that CAN was present in 15% of newly diagnosed T2D cases (7). However, less is known about the progression over time of autonomic neuropathy in individuals with normal glucose tolerance (NGT) and impaired glucose tolerance (IGT) and in patients with T2D diabetes.

There are conflicting data regarding whether or not metabolic factors other than diabetes influence the development of autonomic neuropathy. Body mass index (BMI) has been associated with CAN in T2D in some studies (7, 8), but not in all (9).

In this study, we aimed to investigate the prevalence of autonomic neuropathy and its temporal development, measured as *E*/*I* ratio and patient-reported symptoms, in persons with NGT and IGT, and in patients with T2D.

## Materials and Methods

### Study Population

The study population has been described in an earlier publication (10). Briefly, participants were recruited from the Västerbotten Intervention Programme (11) in Skellefteå, Sweden, during 2003 and 2004. Two standardized oral glucose tolerance tests (OGTT) were performed to determine whether the individuals had NGT or IGT. T2D patients were recruited from their respective primary care centers in the same area during the same time period. At follow-up, two OGTT were performed on both the participants with NGT and those with IGT.

All participants gave their signed, informed consent, and the study was approved by the Regional Ethics Board in Umeå, Sweden.

### *E*/*I* Ratio

Expiration/inspiration ratio (*E*/*I*) as a measure of autonomic function was tested in order to detect any abnormalities. *R*–*R* variation, which is used to calculate the *E*/*I* ratio, is the measurement of sinus arrhythmia (5). The sinus arrhythmia that is seen in healthy individuals when breathing (and is exaggerated in deep breathing) is a result of cyclic variations in sympathetic and parasympathetic tone that modulate the rate of the sinoatrial node (12). In patients with diabetes and autonomic dysfunction, the heart rate variability is reduced or absent (13). Using standard ECG recording, *R*–*R* intervals were analyzed during deep inspiration and expiration (over 1 min at a frequency of 6 breaths/min). The mean value of inspirations and expirations, in terms of *R*–*R* intervals, was used to calculate the expiration: inspiration ratio (*E*:*I* ratio) (13).

### Abnormal *E*/*I* Ratio

The 2004 NGT group was chosen as reference, with a mean *E*/*I* ratio of 1.27 (95% CI 1.21–1.32). An *E*/*I* ratio of <1.5 SD of the mean for the NGT group was considered abnormal (14).

### Symptoms of Autonomic Nerve Function—Autonomic Symptom Score (ASS)

A 7-item questionnaire was used to assess whether the participants had autonomic symptoms (14). The items were as follows: postural hypotension, urinary incontinence, nocturnal diarrhea, gustatory sweating, gastric atony, hypoglycemic unawareness, and erectile dysfunction. The questions were scored according to how often the patient experienced the symptoms: 0 = never, 1 = sometimes, or 2 = often. The scores were then added to give a total ASS.

### Blood Samples

Fasting blood samples were drawn both at baseline and at follow-up, and included HbA1c, S-cholesterol, triglycerides, high-density lipoprotein (HDL), and low-density lipoprotein (LDL).

### Statistics

Statistical calculations were performed using IBM SPSS Statistics, version 22 (SPSS Inc., Chicago, IL, USA). Variables normally distributed are presented as mean ± SD. Non-normally distributed variables are presented as median [interquartile range].

Wilcoxon signed-ranks test was used to compare differences over time. McNemar was used to analyze difference in proportions over time. To compare groups, we used Kruskal–Wallis and the Bonferroni correction for multiple testing. Linear regression analysis was used to calculate the effect of individual variables on *E*/*I* ratio. Pearson’s correlation test was used to calculate correlation and we considered a *R*^2^ of >±0.3 significant. A *p*-value of <0.05 was considered statistically significant.

## Results

### Study Participants

**Figure 1:**
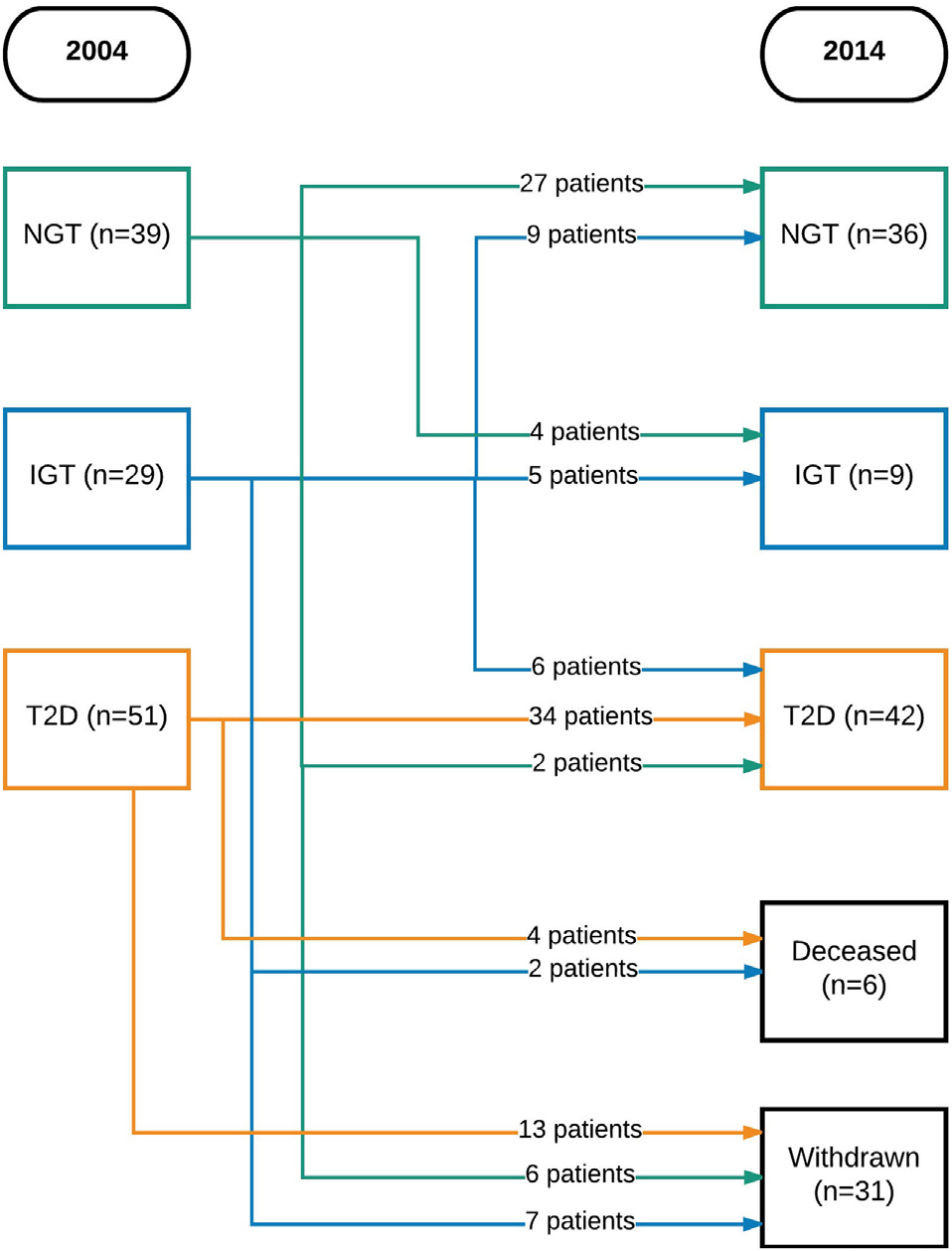
Flowchart of patients over time. NGT, normal glucose tolerance; IGT, impaired glucose tolerance; T2D, type 2 diabetes mellitus.

Initially, 129 participants were included. Of these, six were excluded (three had vitamin B12 or folate deficiency and three had a neurological disease) and four declined to participate. Thus, 119 participants could be included in the study. There were 39 with NGT, 29 with IGT, and 51 with T2D. In 2014, 6 were deceased and 31 chose to withdraw from the study, leaving 87 participants to be included in the follow-up (Figure 1).

### Population Characteristics

The characteristics of the study group (*n* = 87) are presented in Table 1. HbA1c rose over time in those with NGT and IGT, but not in patients with T2D. S-cholesterol, S-HDL, and S-LDL, as well as triglycerides, were higher in 2014 than in 2004 in the total population. The NGT group did not gain weight over the 10 years. More NGT individuals were treated with anti-hypertension agents and lipid-lowering agents in 2014 than in 2004 (Table 1). In patients with T2D, triglycerides and HDL were higher in 2014, whereas LDL cholesterol was lower in 2014 compared to 2004 (Table 1). Baseline characteristics of the study population can be reviewed in Table S5 in Supplementary Material.

**Table 1:** Characteristics of the population comprising healthy persons and patients with impaired glucose tolerance (IGT) and type 2 diabetes (T2D) 2004 and 2014.

	Normal glucose tolerance (NGT) 2004	NGT 2014	IGT 2004	IGT 2014	T2D 2004	T2D 2014	Total 2004	Total 2014
Total (*n*)	33	36	20	9	34	42	87	87
Female, *n* (%)	18 (55)	19 (53)	9 (45)	4 (44)	15 (44)	19 (45)	42 (47)	42 (47)
Body mass index (kg/m^2^)^a^	25.8 (±3.6)	25.5 (±3.6)	27.1 (±5.4)	27.4 (±6.3)	29.7 (±4.3)	29.4 (±4.3)	27.6 (±4.6)	27.5 (±4.6)
HbA1c (mmol/mol)^a^	35 [33–37]	38 [36–40]*	36.5 [34–38]	40 [38–42]*	55 [47–63]^€^	52 [46–64]^€^^,^*	38 [34–52]	42 [38–52]*
S-cholesterol (mmol/L)	5.9 [5.2–6.4]	5.9 [5.1–7.0]*	5.1 [4.3–5.9]	5.9 [4.9–6.05]	4.7 [4.3–5.1]	4.6 [4.2–5.33]^€^	5.1 [4.4–5.9]	5.2 [4.5–6.2]*
S-triglycerides (mmol/L)	1.09 [0.82–1.52]	1.35 [0.84–1.78]*	1.35 [0.85–1.65]	1.41 [1.05–2.51]	1.47 [1.08–1.99]	2.2 [1.58–2.82]^€^^,^*	1.34 [0.95–1.70]	1.73 [1.09–2.37]*
S-high-density lipoprotein (mmol/L)	1.38 [1.2–1.66]	1.41 [1.27–1.70]	1.15 [1.01–1.54]	1.17 [0.88–1.69]	1.10 [0.81–1.37]	1.28 [1.0–1.43]^€^^,^*	1.22 [1.04–1.47]	1.33 [1.09–1.53]*
S-low-density lipoprotein (mmol/L)	3.9 [3.3–4.4]	3.9 [3.2–4.7]*	3.1 [2.08–3.85]	3.5 [2.9–3.95]	2.80 [2.38–3.25]	2.5 [2.03–2.98]^€^	3.2 [2.5–4.0]	3.2 [2.4–4.3]^ns^
Oral hypoglycemic agent,^b^*n* (%)	0 (0)	0 (0)	0 (0)	0 (0)	19 (56)	26 (62)	19 (22)	26 (30)
Insulin treatment, *n* (%)	0 (0)	0 (0)	0 (0)	0 (0)	9 (26)	14 (33)	9 (10)	14 (16)
Beta blocker treatment, *n* (%)	7 (21)	3 (8)	4 (20)	4 (44)	6 (18)	11 (26)	17 (20)	18 (21)
Anti-hypertension agents, *n* (%)	11 (33)	16 (44)	7 (35)	4 (44)	19 (56)	31 (74)	37 (43)	51 (59)
Lipid-lowering agents, *n* (%)	3 (9)	6 (17)	3 (15)	1 (11)	17 (50)	22 (52)	23 (26)	29 (33)

*^a^Data already published (29)*.

*^b^Data missing in one patient*.

*p < 0.05; 2004 vs. 2014, Wilcoxon.

*^€^p < 0.05, comparison between groups, Kruskal–Wallis adjusted by the Bonferroni correction for multiple tests. The significant differences were found between NGT and T2D*.

*ns, non-significant*.

*Values are mean ± SD or median [25th–75th percentiles; i.e., IQR]*.

### Autonomic Symptom Score

The ASS rose over time in the whole population (Table 2). The NGT group showed almost no difference at all in their ASS (mean difference between 2004 and 2014 = 0.06), while the difference was higher in the T2D patients (mean difference between 2004 and 2014 = 0.9; *p* < 0.05) between 2004 and 2014 (Table 2). Results from the individual questions are shown in Table 3.

**Table 2:** Autonomic symptom score (ASS) and expiration/inspiration ratio at baseline (2004) and follow-up (2014).

	Normal glucose tolerance (NGT) 2004	NGT 2014	Impaired glucose tolerance (IGT) 2004	IGT 2014	Type 2 diabetes (T2D) 2004	T2D 2014	Total 2004	Total 2014
ASS	0.79 ± 0.70	0.83 ± 1.12	0.60 ± 0.68	1.11 ± 0.93	1.21 ± 1.30	1.98 ± 1.59^€^	0.91 ± 1.0	1.42 ± 1.45*
Delta ASS		0.06 ± 0.80		0.33 ± 1.12		0.90 ± 1.53		0.5 ± 1.29^€^
*E*/*I* ratio	1.24 [1.17–1.34]	1.17 [1.07–1.24]*	1.17 [1.09–1.30]	1.24 [1.10–1.74]	1.19 [1.11–1.32]	1.13 [1.07–1.22]*	1.21 [1.13–1.32]	1.14 [1.07–1.24]*
Abnormal *E*/*I* ratio	0	3	0	0	0	5	0	8^#^
Delta *E*/*I*		−0.12 [−0.2 to 0.0]		0.02 [−0.10 to 0.48]		−0.06 [−0.18 to 0.0]		−0.07 [−0.18 to 0.0]^ns^

*p < 0.05; 2004 vs. 2014, Wilcoxon.

*^#^p < 0.05; 2004 vs. 2014, McNemar*.

*^€^p < 0.05, comparison between groups, Kruskal–Wallis adjusted using the Bonferroni correction for multiple tests. A significant difference was found between NGT and T2D*.

*ns, non-significant*.

*Values are mean ± SD or median [25th–75th percentiles; i.e., IQR]*.

**Table 3:** Presence of autonomic symptoms in the study population at follow-up (2014).

	NGT (*n* = 36)	Impaired glucose tolerance (*n* = 9)	Type 2 diabetes (T2D) (*n* = 42)	*p*-Value
Postural hypotension, *n* (%)	15 (42)	4 (44)	25 (60)	ns
Urinary incontinence, *n* (%)	1 (3)	1 (11)	7 (28)	ns
Nocturnal diarrhea, *n* (%)	1 (3)	0 (0)	0 (0)	ns
Gustatory sweating, *n* (%)	0 (0)	0 (0)	6 (14)	<0.05
Gastric atony, *n* (%)	2 (6)	0 (0)	5 (12)	ns
Hypoglycemic unawareness, *n* (%)	3 (8)	1 (11)	13 (31)	<0.05
Erectile dysfunction, *n* (%)	6 (17)	3 (33)	11 (26)	ns

*Chi-square was used to calculate statistical significance*.

*ns, non-significant*.

Thirteen out of 42 (31%) patients with T2D reported hypoglycemia unawareness (Table 3). Of these, nine received insulin treatment, one was treated with two oral agents, two patients did not have any anti-diabetic medication and data were missing for one patient. Two of the 13 (15%) T2D patients who reported hypoglycemia unawareness had an abnormal *E*/*I* ratio.

Six (14%) T2D patients reported gustatory sweating. Of these, one had an abnormal *E*/*I* ratio. Urinary incontinence was reported in 7 (28%) T2D patients, and one of these had an abnormal *E*/*I* ratio. Erectile dysfunction was reported in 11 (26%) T2D patients, and 1 of these had an abnormal *E*/*I* ratio. Gastric atony was reported in five (12%) T2D patients, one had an abnormal *E*/*I* ratio (Table 3). Nocturnal diarrhea was not analyzed further, since it was only present in one patient with NGT.

There were no differences in blood pressure levels between individuals who reported postural hypotension and those who did not.

### Autonomic Testing—*E*/*I* Ratio

The rate of abnormal *E*/*I* ratio in the T2D group was 12% (5/42). No differences were found in HbA1c, BMI, ASS, duration of diabetes, or age between patients with abnormal *E*/*I* ratios and those with normal *E*/*I* ratios (data not shown). The *E*/*I* ratio for the population as a whole deteriorated over the study period (Table 2). No statistically significant differences were found in *E*/*I* ratios between groups in 2004 or in 2014. The change in *E*/*I* ratio (i.e., delta *E*/*I*) did not differ between groups. The *E*/*I* ratio did not correlate with the ASS.

Two individuals with abnormal *E*/*I* ratios in 2014 were treated with beta blockers (one in the NGT group and one in the T2D group).

### *E*/*I* Ratio and HbA1c

**Figure 2:**
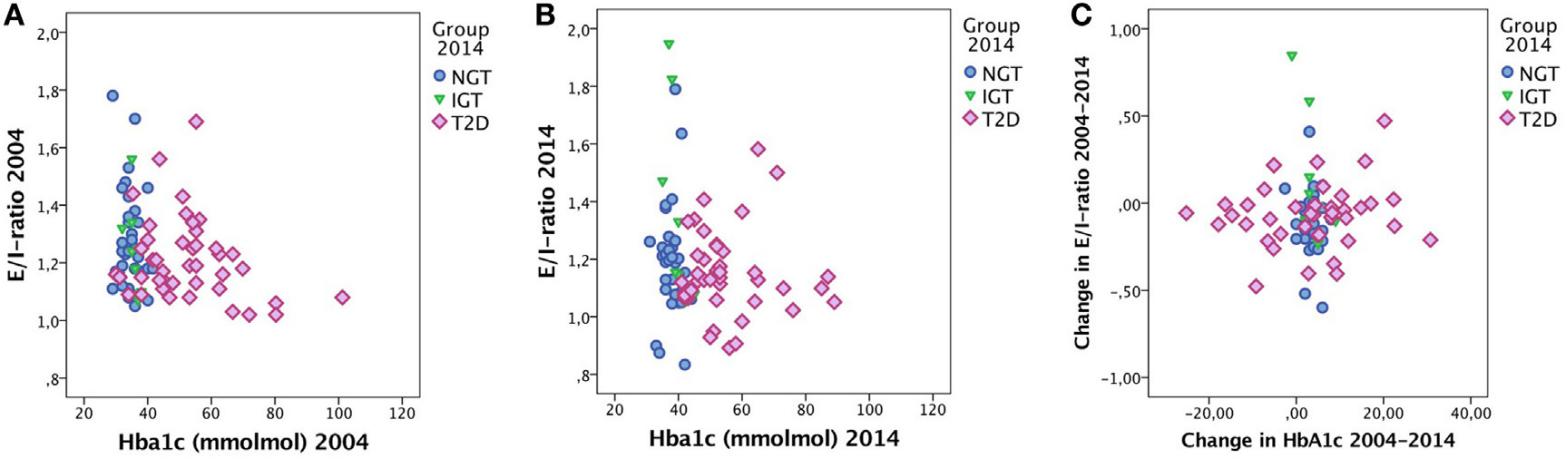
*E*/*I* ratio and HbA1c in **(A)** 2004 and **(B)** 2014 and **(C)** the change in *E*/*I* ratio over time vs. the change in HbA1c over time. Cases labeled by groups [normal glucose tolerance (NGT), impaired glucose tolerance (IGT), and type 2 diabetes (T2D)].

No correlation was found between change in *E*/*I* ratio and change in HbA1c. Furthermore, no correlation was found between *E*/*I* ratio and HbA1c. Figure 2 shows the relationship between *E*/*I* and HbA1c based on groups. In the regression analysis, HbA1c levels were not associated with *E*/*I* ratio.

### Duration of Diabetes

There was no correlation between the duration of diabetes and *E*/*I* ratio, nor between duration of diabetes and ASS symptoms.

### Body Mass Index

In the regression analysis, we found that BMI was the only factor that influenced the *E*/*I* ratio in the T2D patients (Table 4). We did not see any effect of BMI on *E*/*I* ratio in the NGT group (Table 4).

**Table 4:** Linear regression analysis.

	Model 1 T2D	Model 1 NGT
Age	−0.002 (−0.053 to 0.049)	−0.003 (−0.128 to 0.122)
Male gender	−0.02 (−0.140 to 0.099)	0.105 (−0.067 to 0.277)
BMI	0.02* (0.006 to 0.033)	0.016 (−0.002 to 0.034)
HbA1c	−0.003 (−0.008 to 0.001)	−0.009 (−0.038 to 0.021)
Beta blocker treatment	−0.064 (−0.202 to 0.074)	−0.098 (−0.364 to 0.169)
Duration of diabetes	0.002 (−0.009 to 0.014)	
*N*	33	42

*The effect of individual variables on E/I ratio in 2014 in patients with T2D and individuals with NGT*.

*B-coefficient, 95% confidence interval in brackets*.

*p < 0.05.

*T2D, type 2 diabetes; NGT, normal glucose tolerance*.

## Discussion

Our study shows that the patients with T2D, drawn from well-defined populations of T2D and individuals with normal and IGT, had higher ASSs both at baseline and at the long-term follow-up after 10 years. Furthermore, the increase in ASSs over time was higher in T2D patients. This was in spite of strict glucose control, since HbA1c values did not change significantly in the patients with T2D during the study period. During the time frame of 10 years, the *E*/*I* ratio deteriorated in the population as a whole, but there were no significant differences between the three groups, indicating that the deterioration was most probably an effect of aging (15, 16).

We did not find any correlation between the reported autonomic symptoms, as expressed by the autonomic score, and the *E*/*I* ratio, which is in line with previous findings in T2D (17, 18). Autonomic symptoms in T2D seem to be unspecific and not correlated to an abnormal *E*/*I* ratio. Many of our participants reported symptoms of postural hypotension regardless of glycemic status. The explanation for this might be that many factors, other than autonomic dysfunction, influence the blood pressure response to standing up, such as anti-hypertension medication, which was present in many of our patients. Hypoglycemia unawareness was reported by some individuals in the NGT group—the most plausible explanation for this is that there might be a misinterpretation of hypoglycemia unawareness. In the DCCT trial, the number of patients with T1D reporting hypoglycemia unawareness was higher in the intensive treatment group (5). Furthermore, in this study, the majority of T2D patients reporting hypoglycemia unawareness were on insulin treatment.

Erectile dysfunction is an unreliable marker of autonomic neuropathy since the pathogenesis of such symptoms is multifactorial and may be related to aging (19). Hypoglycemia unawareness and gustatory sweating are considered more reliable symptoms in the diagnosis of autonomic dysfunction. They probably appear later in the disease trajectory and indicate more severe nerve damage (20). However, as Vinik et al. conclude in their review from 2003 (19), there is conflicting evidence regarding the association between autonomic dysfunction and hypoglycemia unawareness. In our data, very few of the patients who reported autonomic symptoms actually had an abnormal *E*/*I* ratio, which suggests that the correlation is weak between the *E*/*I* ratio and symptom manifestations. We do, however, see that the T2D patients experience more autonomic symptoms over time, whereas the individuals with NGT did not increase their ASS. As we have no data on individual parameters of the ASS from baseline, these figures are not presented.

There is an ongoing debate about how to measure and diagnose CAN. Ewing et al. suggested the Ewing battery of tests in 1985, which included blood pressure response to standing and sustained handgrip as well as heart rate response to standing (30:15 ratio), deep breathing (*E*/*I* ratio) and Valsalva maneuver (21). Since then, several suggestions for simplifying the tests have been published. Some authors advocate the use of the 30:15 ratio as the most simple and accurate test (22) while others promote the deep breathing test (23). The Toronto Expert Group recommends that at least two tests should be used in order to reach a definitive diagnosis of CAN, but argues that the presence of one abnormal test can identify early CAN (15). We used the *E*/*I* ratio to detect the presence of autonomic neuropathy, since it is easily reproduced and can be used in a primary care setting as no advanced equipment is needed. Only 12% of our T2D patients had an abnormal *E*/*I* ratio at follow-up, which is slightly lower than previously reported in patients with long-standing T2D (6, 24). This might be because the T2D patients in this study are well-defined and intensively treated, as can be seen from their low and stable HbA1c levels. There is probably also both a healthy volunteer bias and a survival bias in our study.

The *E*/*I* ratio in our study had a wide distribution, independent of glycemic status. This suggests that there is a natural variation in *E*/*I* ratio even in a healthy population. Perhaps the best measure of autonomic deterioration is the drop in *E*/*I* ratio over time. However, there was no significant difference between groups when comparing the change in *E*/*I* ratio, perhaps because the changes demonstrated were very small, even after 10 years.

Traditionally, the *E*/*I* ratio has been thought of as a measure of primary parasympathetic function (13). However, newer research suggests a delicate sympathovagal balance behind the variability in heart rate during deep breathing (25, 26). The discussion concerning these delicate mechanisms is outside the scope of the present study, but has been highlighted by other authors with more sophisticated methods than used in the present paper (27).

In future prospective studies, it would probably be wise to use more than one method for measuring autonomic neuropathy, in order to increase sensitivity in identifying affected individuals. We would like to suggest blood pressure response to standing as an additional test, since it is simple, non-invasive, and can be used in basically any setting. We would also like to argue that a symptom-scoring system should be used and that self-reported symptoms might be of more use to the clinician than the results of the autonomic tests in providing the best possible treatment for diabetes patients. When designing future symptom-scoring questionnaires, we suggest that instead of asking about erectile dysfunction a more gender neutral question could be used to evaluate the prevalence of both male and female sexual dysfunction. Complex questions have been used in earlier studies (28). We suggest asking whether the responder is experiencing any sexual dysfunction, whether it started recently and whether they would like to specify the dysfunction.

Regarding metabolic factors, we found a positive association between *E*/*I* ratio and BMI in the T2D patients. This contrasts with previous studies where the association has been in the other direction (7, 8). The effect in this study is, however, very small and may be a chance effect, rather than a true effect, and thus of no clinical significance.

In the study design, we included patients with IGT. At follow-up, this group was very small, and we have therefore chosen not to focus on these individuals.

### Strengths and Limitations

The study group is well defined and the follow-up time of 10 years is long. All study participants were evaluated using the same methods, carried out by the same examiner (the same physician both at baseline and at follow-up), in order to ensure that each participant experienced exactly the same test conditions, thereby reducing potential confounding factors (15). Our study is probably prone to survival bias and healthy participant bias, since 4 of the T2D patients (7%) passed away during the study period and 31 patients (25%) withdrew from the study; many of them probably due to poor health. This is a population with an expected high mortality due to age and co-morbidity. Moreover, the questionnaire did not include a question regarding female sexual dysfunction, so only half of the population could report on symptoms of sexual dysfunction.

## Conclusion

The presence of autonomic symptoms rises over time in T2D patients, but such symptoms do not correlate with the *E*/*I* ratio in a well-defined cohort. Autonomic symptom scorings can be a useful clinical tool when assessing the progression of autonomic dysfunction in patients with abnormal glucose metabolism.

## Ethics Statement

All participants gave their signed informed consent, and the study was approved by the Regional Ethics Board in Umeå, Sweden.

## Author Contributions

OR initiated the study. MZ made the calculations and wrote the manuscript. All authors contributed to the discussion and to revising the manuscript.

## Conflict of Interest Statement

The research was conducted in the absence of any commercial or financial relationship that could be construed as a potential conflict of interest.
