# Supplementary material for: Autonomic Neuropathy—a Prospective Cohort Study of Symptoms and E/I Ratio in Normal Glucose Tolerance, Impaired Glucose Tolerance, and Type 2 Diabetes
Source: Front Neurol. 2018 Mar 14;9:154. doi: 10.3389/fneur.2018.00154 (PMC5861181; doi:10.3389/fneur.2018.00154)
Supplement: Supplementary file 1 [file Table_5.PDF]

Supplementary material

Table 5. Baseline characteristics of the study population by groups

	NGT 2004 (n=39)	IGT 2004 (n=29)	T2D 2004 (n=51)	p-value Kruskal Wallis	Total (n=119)
Women, n (%)	20 (50)	14 (48)	24 (42)		55 (46)
Age	61±0.6	61±0.8	61±1.3		61±1.0
BMI	26±3.6	27±5.4	30±4.6	ns	28±4.7
E/I ratio	1.24 [1.17-1.34]	1.18 [1.1-1.25]	1.18 [1.08-1.27]	ns	1.19 [1.13-1.29]
Abnormal E/I ratio	0	0	0		0
HbA1c (mmol/mol)	35 [33-37]	36 [34-38]	55 [43-66]	<0.0001*	38 [34-54]
ASS score	0.77±0.71	0.93±1.1	1.20±1.4	0.003**	0.99±1.1

*significant between NGT-T2D and IGT-T2D
**significant between NGT-T2D
ns = non-significant
